# Bacteriophage as a novel therapeutic approach for killing multidrug-resistant *Escherichia coli* ST131 clone

**DOI:** 10.3389/fmicb.2024.1455710

**Published:** 2024-12-12

**Authors:** Md Shamsuzzaman, Shukho Kim, Jungmin Kim

**Affiliations:** ^1^Department of Biomedical Science, The Graduate School, Kyungpook National University, Daegu, Republic of Korea; ^2^Department of Microbiology, School of Medicine, Kyungpook National University, Daegu, Republic of Korea

**Keywords:** *Escherichia coli*, clone ST131, antibiotic resistance, bacteriophages, therapeutic efficiency, phage cocktail

## Abstract

The emergence of the multidrug-resistant (MDR) *Escherichia coli* ST131 clone has significantly impacted public health. With traditional antibiotics becoming less effective against MDR bacteria, there is an urgent need for alternative treatment options. This study aimed to isolate and characterize four lytic phages (EC.W2-1, EC.W2-6, EC.W13-3, and EC.W14-3) from hospital sewage water and determine their effectiveness against the ST131 clone. These phages demonstrated a broad host range, effectively lysing 94.4% of highly pathogenic *E. coli* ST131 isolates. Morphological observations and phylogenetic analysis indicate that EC.W2-1, and EC.W13-3 belong to the *Tequatrovirus* genus in the *Straboviridae* family, while EC.W2-6 and EC.W14-3 are part of the *Kuravirus* genus in the *Podoviridae* family. Phages remained stable at pH 2–10 for 4 h and below 80°C for 1 h. These four phages showed *in vitro* bacterial lytic activity at various MOIs (0.1–0.001). The one-step growth curve of phages exhibited a short latent period of approximately 10–20 min and a moderate burst size of 50–80 (pfu/cell). Phages’ genome size ranged from 46,325–113,909 bp, with G + C content of 35.1 –38.3%. No virulence or drug resistance genes were found, which enhanced their safety profile. *In vivo*, EC.W2-6 and EC.W13.3, along with their cocktail, fully protected against the ESBL-producing *E. coli* ST131 infection model *in vivo*. Combining these phages and a 3-day repeated single phage, EC.W13-3 significantly enhanced the survival rate of *E. coli* ST131 infected mice at low MOI (0.01–0.001). The *in vivo* effectiveness of the isolated phages and the EC.W2-6 and EC.W14-3 cocktail in highly reducing bacterial load CFU/g in multiple organs strongly supports their potential efficacy. Based on *in vivo*, *in vitro*, and genomic analyses, phages have been proposed as novel and suitable candidates for killing the pandemic ST131 clone.

## 1 Introduction

*Escherichia coli* sequence type 131 (ST131) is a clonal group of concern, known for causing antimicrobial-resistant infections predominantly in community settings (Rogers et al., 2011). The ST131 pandemic was initially detected in 2008 using multilocus sequence typing (MLST) to identify CTX-M-15 extended-spectrum *β*-lactamase-producing *E. coli* strains across various continents. *E. coli* ST131 is mostly known as a major cause of urinary tract infections (UTIs). Besides causing UTIs, this extraintestinal pathogenic bacterium can also lead to serious infections, including bloodstream infections, sepsis, and intra-abdominal infections (Whelan et al., 2023). These infections affect individuals of all ages and range from cystitis to life-threatening sepsis. Treatment requires a tailored approach considering antimicrobial resistance and infection site. Although phenotypic detection of ST131 is not possible, DNA-based methods like MLST and PCR are used for identification (Rogers et al., 2011; Nicolas-Chanoine et al., 2014).

The clone ST131, which is a major cause of fluoroquinolone-resistant *E. coli* infections, has a high prevalence ranging from 60 to 90% (Peirano et al., 2020). Fluoroquinolone resistance in ST131 is caused by amino acid substitutions in the QRDR genes (*gyrA, parC*, and *parE*), which reduce the effectiveness of fluoroquinolone in inhibiting bacterial DNA replication and transcription (Shariati et al., 2022). Recently, the plasmid-mediated colistin resistance gene *mcr*-1 was detected in ST131. The presence of *mcr*-1 in ST131 raises concerns about the spread of colistin resistance (Majewski et al., 2021). The ST131 clone of *E. coli* harbors different *β*-lactamase genes (Miyoshi-Akiyama et al., 2016). *β*-lactamases are enzymes produced by bacteria that can deactivate *β*-lactam antibiotics such as penicillins and cephalosporins. The most common types of *β*-lactamase genes in the ST131 clone are CTX-M family *β*-lactamases, with TEM, SHV, and CMY *β*-lactamases being less frequent. Approximately 40–80% of extraintestinal pathogenic *E. coli* strains with extended-spectrum *β*-lactamases (ESBLs) belong to the ST131 clone (Hibstu et al., 2022).

The geographical distribution of ST131 is not fully known; however, it is commonly found in antimicrobial-resistant *E. coli* infections in Europe, North America, Canada, Japan, and South Korea (Bevan et al., 2017). In a national survey in Japan, the ST131 clones were found in 38.1% of ESBL-producing *E. coli* isolates between 2013 and 2014. Notably, in this region, there is higher genetic diversity within the ST131 clone and a greater variety of accompanying CTX-M ESBL genes than in other areas (Miyoshi-Akiyama et al., 2016). Similarly, in South Korea, from 2016 to 2017, 27% of ESBL-producing *E. coli* isolates were ST131, with only 57% carrying an ESBL gene (Baek et al., 2021). *E. coli* ST131, an antimicrobial-resistant clone, has been found in humans, companion animals, non-companion animals, and food sources. The extensive presence of ST131 in various populations raises concerns regarding its potential transmission and its impact on public health (Banerjee and Johnson, 2014).

As mentioned previously, the ST131 clone of *E. coli* possesses various antimicrobial resistance mechanisms. However, limited information is available regarding the specific antimicrobial therapies used to treat the infections caused by this clone. This situation underscores the pressing need for innovative therapeutic approaches and development of new antimicrobial agents to effectively combat these infections (Kim et al., 2018; Rehman et al., 2019). The limited availability of new antibiotics has renewed interest in phage therapy as a potential solution, increasing the search for novel phages (Reardon, 2014). Phages are viruses that infect and kill bacteria, offering a potential alternative to antibiotics in the face of growing antimicrobial resistance. Clinical approaches to phage therapy have been reported in several countries, including the United States, Georgia, Poland, and Russia (Hibstu et al., 2022). Phages exhibit selective tropism, targeting specific bacteria via surface receptors while minimizing harm to the host microbiome. Generally safe for mammals, potential risks include immune responses and endotoxin release. Lytic phages directly kill bacteria and can be engineered for targeted drug delivery (Emencheta et al., 2024). Scientific evidence supporting the benefits of phage therapy in humans and animals has steadily increased in the recent decades (Hon et al., 2022). Additionally, they have been used as indicators of water safety (de Almeida Kumlien et al., 2021). As the availability of phages is limited, there is an urgent need to isolate safe, highly lytic, and well-characterized phages for phage therapy.

Current research focuses on isolating, characterizing, and evaluating the efficacy of novel *E. coli* phages in combating ST131 *E. coli* infections *in vitro* and *in vivo*. The goal of this study was to explore phages as an alternative strategy to combat the growing issue of MDR *E. coli* clone ST131. However, phage therapy is still in its early stages and faces challenges, such as regulatory barriers and manufacturing limitations. Nonetheless, advancements in phage therapy have provided hope for addressing antibiotic resistance and improving the treatment options for serious bacterial infections.

## 2 Materials and methods

### 2.1 Animals used in *in vivo* experiments

*In vivo* experiments were conducted on BALB/c mice strain from Yeangnam Bio (166 Palgong-ro, Dong-gu Daegu) with six-week-old female mice receiving sterile food and water. The procedures followed guidelines set by the National Ethics Committee and approved by the Kyungpook National University Animal Care and Use Committee (KNU: 2023–0478).

### 2.2 Bacterial strains and growth conditions

*E. coli* isolates were obtained from Kyungpook National University Hospital Culture Collection for Pathogens (KNUH-NCCP). The isolates were initially cultured on 5% sheep blood agar at 37°C for 24 h. Then, the samples were transferred to Brain Heart Infusion broth (BHI) and incubated at 37°C for 24 h. Finally, the isolates were preserved at −70°C with a 50% glycerol stock for future experiments.

### 2.3 Phage isolation, purification, and preparation

*E. coli* hosts were cultured on blood agar plates. Sewage water samples were collected from Kyungpook National University Hospital in Daegu, South Korea. Sewage water was treated by centrifugation at 12,000 × g for 10 min to remove the debris. The resulting supernatant was filtered using 0.22 μm pore-sized membrane filters. The filtrate was used to infect *E. coli* cultures in the early exponential phase. After overnight incubation at 30°C with shaking, infected cultures were stored at 4°C for 48 h. The supernatants were centrifuged again and filtered through a 0.22 μm pore-size membrane. Phage titers were determined using the double layer method. The purified phages were stored at −70°C in glycerol-supplemented medium (Kim et al., 2022).

### 2.4 Antimicrobial susceptibility test and MLST analysis

Antibiotic susceptibility of the 18 *E. coli* isolates was tested against 21 antibiotics from 10 families. These families include cephalosporins, monobactams, aminopenicillin, fluoroquinolones, aminoglycosides, penicillin, sulfonamide-trimethoprim, tetracycline, carbapenem, and extended-spectrum beta-lactamases. To determine the minimum inhibitory concentration (MIC), antibiotic disks and culture materials were purchased from Becton Dickinson and Company. *E. coli* ATCC25922 was used as the control. Each isolate was tested in triplicate, according to the Clinical and Laboratory Standards Institute guidelines (CLSI, 2020; Shamsuzzaman et al., 2023). According to these guidelines, isolates were classified as resistant, intermediate, or susceptible. The MLST analysis described in our study was previously conducted by the Department of Microbiology at Kyungpook National University (Shamsuzzaman et al., 2024).

### 2.5 Determination of lytic activity of phages against clinical isolates of *Escherichia coli* ST131

Eighteen *E. coli* clinical isolates (ST131) were tested for susceptibility to phage lytic activity by spot tests following the method outlined by Kim et al. (2018). To prepare the bacterial lawn, 10 mL of 0.75% soft agar with 100 μL of bacterial culture in the stationary phase was poured onto a BHA agar plate. Once the overlay solidified, a 15 μL aliquot of the phage, with a concentration of approximately 10^11–13^ PFU/ml, was directly spotted on the bacterial lawn. The plates were dried and incubated overnight at 37°C. Bacterial susceptibility was determined using the clear lytic zones on the plates.

### 2.6 Measurement of adsorption rate and burst size of four novel *Escherichia coli* phages

Phage adsorption rate and burst size were determined following the method outlined by Rahman et al. (2011) *E. coli* cells (ATCC25922) were cultured in BHI media until they reached exponential phase. The cells were then infected with the phage at a low multiplicity of infection (MOI) of 0.0001 and incubated at room temperature. Samples were collected and centrifuged at intervals of 0, 1, 3, 5, 10, 15, and 20 min. The supernatants were used for plaque assays to determine the titers of the unabsorbed phages. The burst size of the phage was determined using a single growth-curve experiment. *E. coli* cells were harvested by centrifugation and resuspended in 1 mL of fresh BHI medium at a concentration of 5 × 10^9^ CFU/mL. The phage was added at an MOI of 0.0001 and allowed to adsorb for 30 min at 4°C. After centrifugation at 12,000 × g for 5 min, the pellet was resuspended in 10 mL of fresh BHI medium. The mixture was then incubated at 37°C, and samples were collected at 5-min intervals for up to 45 min. The samples were promptly diluted and analyzed for phage plaque counts. Each experiment was independently repeated thrice.

### 2.7 Thermal and pH stability

The thermal and pH stability of the phage were evaluated following the method described by Tang et al. (2023). To assess thermal stability, the phage suspension (∼10^8^ PFU/mL) was incubation at temperatures ranging from 4 to 80°C for 1 h. The phage suspension (∼10^8^ PFU/mL) was incubated in BHI broth with pH levels ranging from 2 to 10 at 37°C for 4 h to determine pH stability. Phage titers were determined utilizing the double-layer agar method.

### 2.8 *In vitro* bactericidal activity at various MOIs and phage cocktail analysis

This method determines the bactericidal activity of phages and phage cocktails against *E. coli* ATCC 25922 and *E. coli* ST131. Bacterial cultures (10^8^ CFU/mL) were treated with individual phages and combination phages (1:1) at various MOIs (10, 1, 0.1, 0.01, 0.001) in BHI broth at 37°C with gentle shaking. Optical density at 600 nm was measured using a UV–Vis spectrophotometer (Molecular Devices, LLC, San Jose, CA, United States) in a 96-well plate at 1 h intervals for 12 h and again at 24 h. Bacterial culture without phages served as the positive control, while BHI broth served as the negative control. All assays were performed in triplicate (Tang et al., 2023).

### 2.9 Bacteriophage sequencing and genome assembly

Phage DNAs were extracted using the phenol-chloroform method described by Jakočiūnė and Moodley (2018). To conduct next-generation sequencing (NGS), phage genomic DNA was sequenced using an Illumina Miseq platform in San Diego, CA, USA. The sequencing reads were assembled through the Celemics pipeline based in South Korea.^1^ To analyze genome sequence similarity, BLASTn^2^ was used for alignment. The RAST online website^3^ was utilized to predict open reading frames (ORFs), and the predictions were cross-checked and corrected through the NCBI database. Gene function maps were created using a custom program developed in the lab and refined using Geneious Prime 2023.2.^4^ The tRNAscan-SE v.2.0 tool^5^ was employed to predict tRNA. ResFinder^6^ and VirulenceFinder^7^ were utilized to identify drug resistance genes and virulence genes, respectively. A phylogenetic tree based on the phage proteome rectangular tree circular tree were generated using VIPTree.^8^ For the construction of a complete genome phylogenetic tree based on the whole genome sequence of the isolated phages, the genome-BLAST distance phylogenetic approach provided by VICTOR^9^ was used. Phylogenetic trees of large terminase and minor capsid proteins were constructed using the neighbor-joining (NJ) method, and the Bootstrap method was employed to assess the reliability of the phylogenetic trees. Additionally, the complete genome sequences of the isolated phages were aligned with other phages using the BLASTn tool available in the NCBI database. The Mauve algorithm (v2.3.1) was used to visualize the complete genome sequence similarity between the isolated phages and their closest neighbor, *E. coli* phages.^10^

### 2.10 *In vivo* bactericidal efficacy of phages in a murine model of *Escherichia coli* ST131

Overnight bacterial cultures were grown in BHI broth at 37°C with shaking reached an OD of approximately 2. To ensure they were in the logarithmic phase, 100 μL of the culture was reinoculated into 10 mL fresh BHI and incubated at 37°C. Once in the logarithmic phase, cultures were washed twice in 1X PBS. Viable bacteria were quantified by plating serial dilutions on bacteriological agar to calculate CFU. Following this, mice were administered an intraperitoneal injection (IP) of a 50% lethal dose (LD50) of 10^9^ CFU/mL. One-hour post-infection, mice were administered intraperitoneal injections of purified phages at varying plaque-forming units (PFU) in 100 μL of PBS. The infected mice were divided into treatment groups for analysis, with each group containing 5 mice. The control group consisted of infected mice that did not receive any treatment. The phage-treated group included mice infected with *E. coli*-specific phages. The positive control group was treated with PBS, whereas the negative control group was treated with both phages and PBS. Daily survival rates were recorded for each group to evaluate the effectiveness of phage therapy in preventing mortality (Pu et al., 2022).

To determine the colony-forming units (CFU) and plaque-forming units (PFU), mice were euthanized 16–18 h post-infection. Following euthanisation, necropsies were performed under sterile conditions. Each treatment group contained 4 mice. The lungs, kidneys, liver, and spleen were weighed and homogenized in 1X PBS using sterile blades. Subsequently, organ homogenates were serially diluted and plated on LB ampicillin (100 μg/mL) plates, followed by overnight incubation at 37°C. Phage counts were quantified using a soft agar overlay assay (Green et al., 2017).

### 2.11 Statistical analysis

Statistical analyses were performed using GraphPad Prism (version X). Survival analysis was conducted using the Kaplan–Meier method, with differences between groups assessed through the log-rank (Mantel-Cox) test. To compare bacterial loads across different organs, one-way ANOVA was used followed by Tukey’s post-hoc test for multiple comparisons. For phage analysis, data from adsorption and burst size assays were analyzed using unpaired t-tests. Results were considered statistically significant at *p*-values <0.05 and are presented as mean ± standard error of the mean (SEM).

## 3 Results

### 3.1 Host range and EOP of phages

Based on the spot test results, phages EC.W2-1, EC.W2-6, EC.W13-3, and EC.W14-3 exhibited high lytic activity against pathogenic ST131 isolates, lysing 94.44% (17/18) of the isolates (Table 1). These phages also showed lytic activity against 14 different ST types, including 60 multi-drug-resistant *E. coli* isolates, with lytic activity percentages ranging from 50.0 to 56.6% (Shamsuzzaman et al., 2024). However, the efficiency of plating (EOP) values of phages against *E. coli* KBN 10PO7288 (ST131) compared to the reference *E. coli* ATCC25922 were approximately 2.56 × 10^9^, 4.56 × 10^6^, 261.53, and 372.09, respectively (Supplementary Table S1).

**Table 1:** Bacteriolytic activities of isolated bacteriophages to kill *Escherichia coli* ST131 clone.

*E. coli* isolates ST131	ΦEC. W2-1 (PP445228)	ΦEC. W2-6 (PP445229)	ΦEC. W13-3 (PP496997)	ΦEC. W14.3 (PP496998)
KBN10P03440	+	+	+	+
KBN10P03979	+	+	+	+
KBN10PO7282	+	+	+	+
KBN10PO7288	+	+	+	+
KBN10P01569	−	−	+	+
KBN10P03452	+	+	+	+
KBN10P03005	+	+	+	+
KBN10P00128	+	+	+	+
KBN10P00238	+	+	+	+
KBN10P02048	+	+	−	−
KBN10P00547	+	+	+	+
KBN10P05638	+	+	+	+
KBN10P06781	+	+	+	+
KBN10P06658	+	+	+	+
KBN10P02511	+	+	+	+
KBN10P05702	+	+	+	+
KBN10P05883	+	+	+	+
KBN10P01583	+	+	+	+

(+) indicates clear lysis, and (−) indicates no lysis observed.

### 3.2 Antibiotic resistance profile of clinically isolated *Escherichia coli* isolates

**Figure 1:**
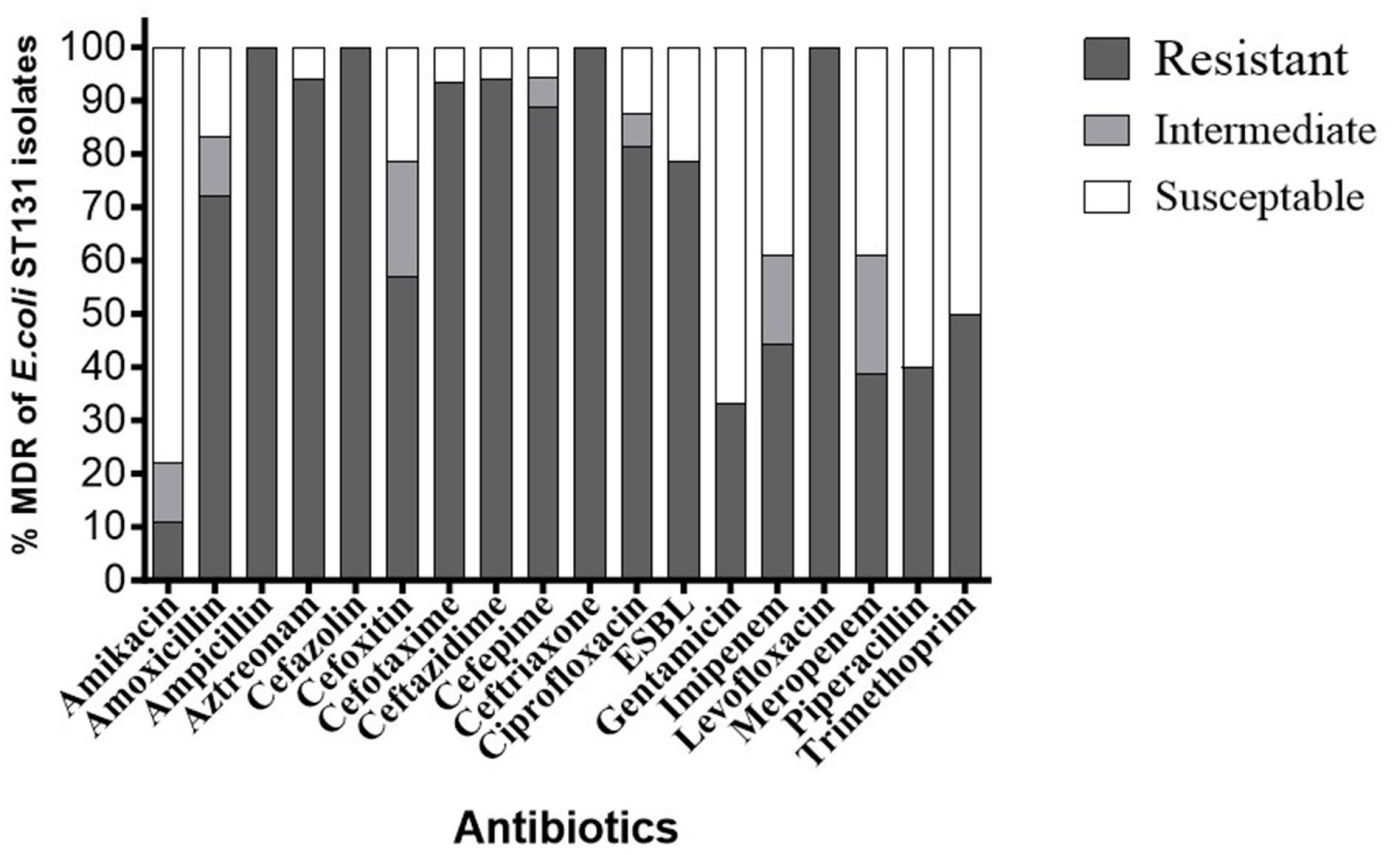
Antibiotic resistance ratios of different *E. coli* ST131 isolates. % Resistance indicates the proportion of isolates of *E. coli* ST131 that resist each antibiotic.

As shown in Figure 1, AST results revealed that all 18 *E. coli* ST131 isolates exhibited a multidrug-resistant profile, indicating an alarming 100% prevalence of MDR (18/18) (Supplementary Table S2). The antibiotic resistance and susceptibility patterns of 18 ST131 isolates were investigated, and results revealed significant resistance to cephalosporins, specifically cefazolin (100%, 14/14), ceftazidime (94.1%, 16/17), cefoxitin (57.1%, 8/14), ceftriaxone (100%, 2/2), cefotaxime (93.3%, 14/15), and cefepime (88.8%, 16/18). The monobactam family also exhibited notable resistance, with aztreonam showing 94.1% resistance (16/17). Aminopenicillins also showed resistant to amoxicillin (72.2%, 13/18) and ampicillin (100%, 12/12), demonstrating high resistance rates. Carbapenem-resistant strains were observed with imipenem (44.4%, 8/18) and meropenem (38.8%, 7/18). The isolates also showed high fluoroquinolone resistance, with 81.2% (13/16) to ciprofloxacin and 100% (6/6) to levofloxacin. The aminoglycoside family showed resistance to amikacin (11.1%, 2/18) and gentamicin (33.3%, 6/18). In addition, the penicillin family resisted piperacillin (40%, 2/5) and the sulfonamide-trimethoprim family resisted trimethoprim (50%, 4/8). ESBL were detected in 78.5% of the isolates (11/14).

### 3.3 Biological and morphological characterization of phages

**Figure 2:**
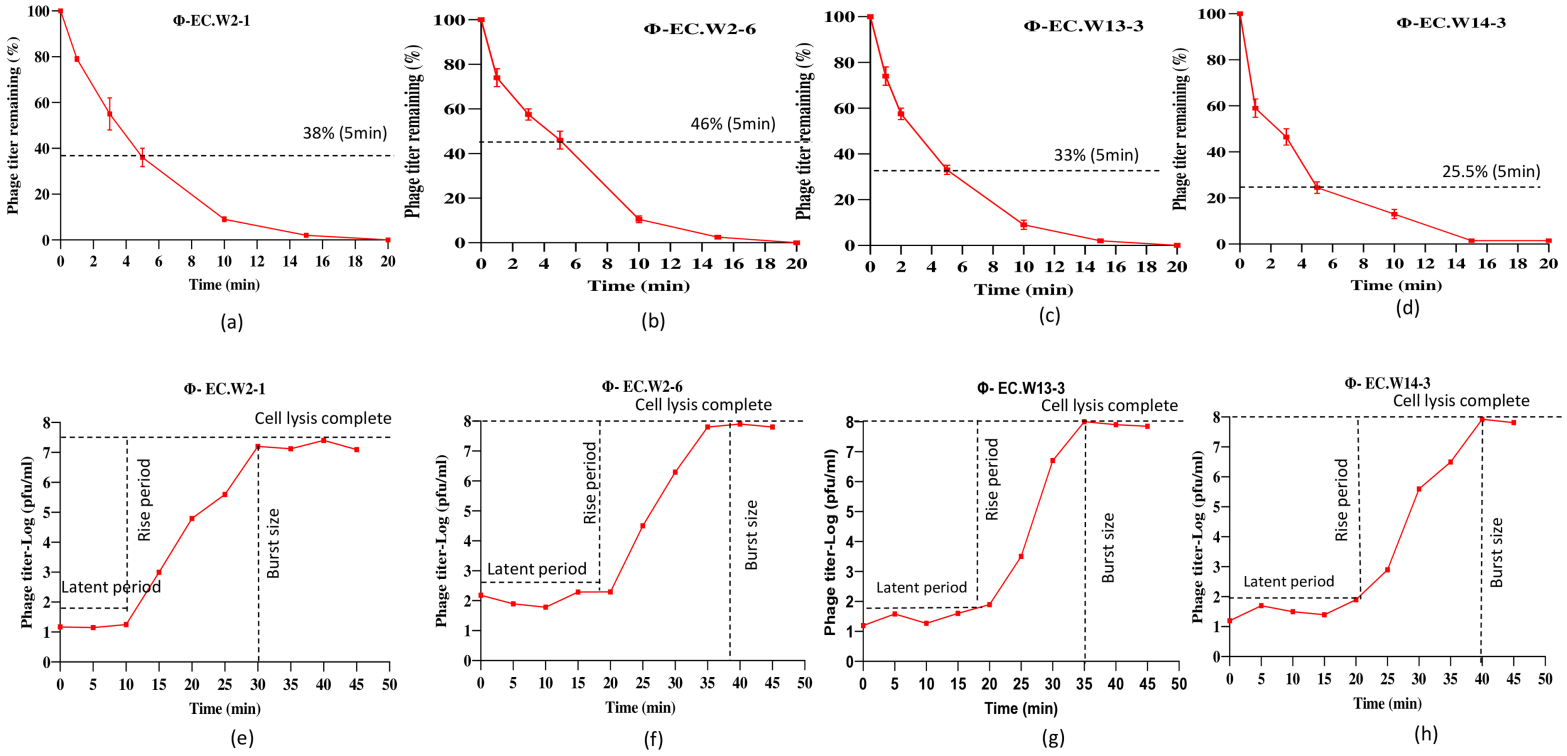
Adsorption rate and burst size of four phages to the *E. coli* ATCC25922. (a–d) Adsorption assay measuring the percentage of remaining free phages over 20 min at MOI of 0.0001 and (e–h) one-step growth curve showing the latent period and burst size of four novel *E. coli* bacteriophages in BHI medium at MOI of 0.0001. Values represent means ± standard deviations from the duplication of each treatment.

**Figure 3:**
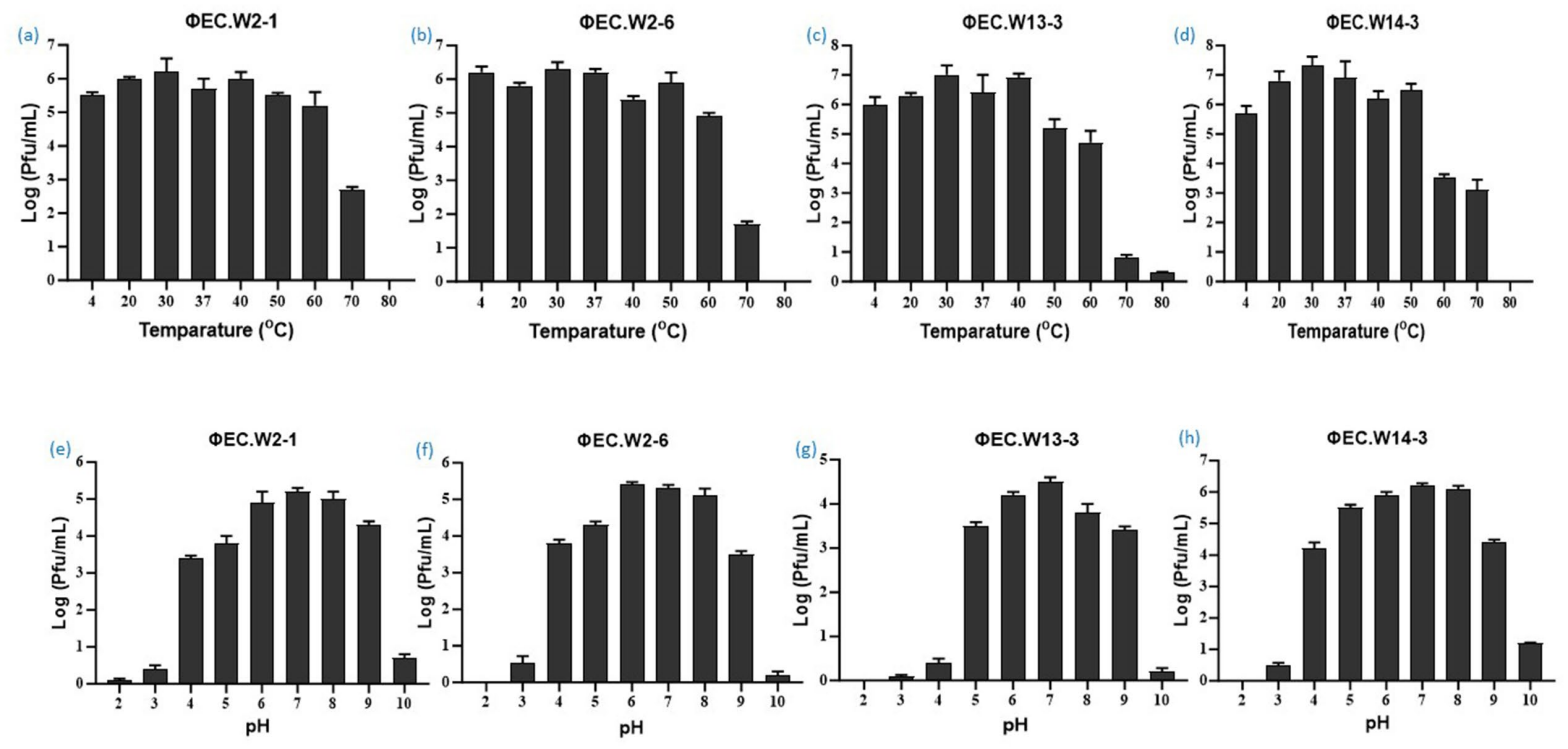
Stability of four novel *E. coli* bacteriophages. (a–d) Effect of temperature on phage stability, with phages treated at 4, 20, 30, 37, 40, 50, 60, 70, and 80°C for 1 h, and surviving phage titers calculated. (e–f) pH stability of phages, with concentrations (~10^8^ PFU/mL) incubated at pH 2 to 10 at 37°C for 4 h. Values represent means ± standard deviations from triplicate treatments.

In this study, we investigated the lytic activity, growth curve, thermal stability, and pH stability of the phages EC.W2-1, EC.W2-6, EC.W13-3, and EC.W14-3. According to the phage adsorption assay, 90% of phages could adsorb onto *E. coli* ATCC25922 within 10 min (Figures 2a–d). The results of the one-step growth curve analysis showed that the phage displayed a latent period of approximately 10–20 min, followed by rapid release of virus particles. The final titer reached a range –10^7.5^ to 10^8^ PFU/mL after a burst period of 30–40 min, with a burst size of approximately 50–80 PFUs/cell (Figures 2e–h). Regarding thermal stability, there were no significant changes in the titers of the four phages after incubation at temperatures ranging from 4 to 60°C for 60 min. However, phage titers showed a substantial decrease at 70°C, and were completely inactivated at 80°C. Furthermore, these phages were stable over a pH range of 2–10 within 4 h, with the optimal pH for stability being between 6 and 8 (Figures 3a–h). Four phages were isolated from the hospital sewage water. After incubation at 37°C for 18 h on a double-layer agar plate, clear plaques with diameters of approximately 1–2 mm. Transmission electron microscopy (TEM) images revealed that EC.W2-1, EC.W13-3, and EC.W14-3 had an icosahedral head measuring 100 ± 2 to 115 ± 5 nm in diameter, along with a contracted tail measuring approximately 100 ± 4 to 113 ± 2 nm in length. In contrast, the EC.W2-6 phage had a head size of 60 ± 3 nm and tail size of 8 ± 2 nm (Shamsuzzaman et al., 2024).

### 3.4 Phylogenetic analysis of phages

The phylogenetic relationship of the novel phages was determined through a comparative analysis of their whole genome sequences with the closest known species of *Straboviridae* and *Podoviridae E. coli* phages. The analysis revealed that EC.W2-1 and EC.W13-3 exhibited the highest DNA sequence similarities to the *Tequatrovirus* genus in the *Straboviridae* family, and phages Escherichia phage vB_EcoM_G4498 (complete genome) and Escherichia phage vB_EcoM_NBG2 (complete genome). For these isolates, the query coverage and percentage identity were recorded as 99/96.45% and 92/95.40%, respectively. On the other hand, EC.W2-6 and EC.W14-3 displayed the highest DNA sequence similarities to the *Kuravirus* genus in the *Podoviridae* family, and Escherichia phage vB_EcoP_SU10 (complete genome) and Escherichia phage 172-1(complete genome), with a query coverage and percentage identity of 94/96.21% and 89/89.62%, respectively (Table 2). Based on the analysis of their sequences and phylogenetic relationships, the phages were identified as new members of the *Straboviridae* and *Podoviridae* families.

**Table 2:** Genomic information of four *E. coli* bacteriophages.

Phage isolates	ΦEC.W2-1	ΦEC.W2-6	ΦEC.W13-3	ΦEC.W14-3
Accession number	PP445228	PP445229	PP496997	PP496998
Class	*Caudoviricetes*	*Caudoviricetes*	*Caudoviricetes*	*Caudoviricetes*
Family	*Straboviridae*	*Podoviridae*	*Straboviridae*	*Podoviridae*
Genus	*Tequatrovirus*	*Kuravirus*	*Tequatrovirus*	*Kuravirus*
G + C	35.5	38.3	35.3	35.1
Genome size (BP)	46,325	67,000	113,909	69,164
Hypothetical protein	31	72	26	20
Phage finder	33	13	71	12
Integration/excision	–	1	–	–
Replication/recombination	3	1	9	1
Stability/transfer/defense	–	–	2	–
Alien hunter	3	2	1	–
Endolysin	–	1	–	–
Holin	–	–	1	–
Tail fiber/spike protein	5	5	5	5
Lysis protein	2	2	3	–
Putative peptidase	–	1	–	1
Internal protein	–	1	–	–
Endonuclease	1	–	–	–
Glutaredoxin	2	–	13	–
Packaging machinery	–	2	–	3
Head protein	1	2	–	–
Thymidylate synthesis	1	1	–	1
Capsid protein	7	–	1	2
Baseplate protein	7	–	6	1
Genomic similarity	NC_054933.1	NC_028903.1	NC_042129.1	NC_027395.1
Query cover/Per. Ident (%)	99/96.4%	88/93.6%	92/95.4%	93/96.21

### 3.5 Genomic feature of four novel *Escherichia coli* phage

**Figure 4:**
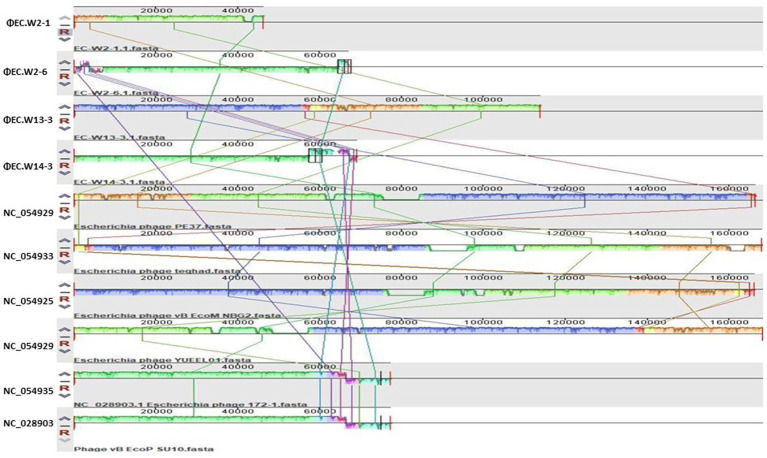
This study employed the progressive Mauve algorithm (v2.3.1) to perform multiple alignments of the genomes of 4 novel *E. coli* bacteriophages and the six closest genomes of *E. coli* bacteriophages. The objective was to investigate the rearrangement patterns and synteny in these genomes. The genomes were laid out horizontally, with homologous segments represented by colored blocks. The regions outside the blocks lacked homology and were represented by white areas, which were unique to each genome and not aligned.

Whole-genome sequence analysis indicated that EC.W2-1, EC.W2-6, EC.W13-3, and EC.W14-3 had circular double-stranded DNA structures, with genome sizes of 46,325, 67,000, 113,909, and 69,164 bp, respectively. Additionally, the G + C contents were found to be 35.5, 38.3, 35.3, and 35.1%, respectively (Table 2). Genome analysis revealed the absence of virulence or drug resistance genes, which enhanced their safety profiles. The CGView server and Geneious Prime generated visual representations of circular genomes, showcasing various aspects such as sequence characteristics, base composition plots, analysis outcomes, and sequences. These visual images provide a comprehensive and intuitive visualization of the genomes, allowing for a better understanding of their structural and compositional features (Supplementary Figures S1a–d). Phylogenetic analysis carried out using the VIPtree showed that EC.W2-1 and EC.W13-3 phages were most closely related to Escherichia phage vB_EcoM_G4498 (NC_054918) Escherichia phage ECO4 (NC_054911), Escherichia phage vB_EcoM_G9062 (NC_054920), Escherichia phage MN03 (NC_070990), and Escherichia phage vB_EcoP-(NC_070989). On the other hand, EC.W2-6, and EC.W14-3 close related to Escherichia phage ECO4 (NC_054911) Escherichia phage 172–1 (NC_028903). All phages belong to the novel genera *Tequatrovirus* and *Kuravirus* within the families *Straboviridae* and *Podoviridae* (Supplementary Figure S2). In addition, alignment with their closest neighbor using the MAUVE alignment tool suggested that these isolates are closely related and share recent common ancestors. However, small localized regions were identified, indicating functional differences between these phages. These findings suggest unique characteristics and capabilities of genetic makeup (Figure 4). Phylogenetic analysis conducted through genome comparisons using the TYGS server also supported EC.W2-1, EC.W2-6, EC.W13-3, and EC.W14-3, which are novel members of the genera *Tequatrovirus and Kuravirus* (Supplementary Figure S3). Analysis of the genome sequences of EC.W2-1, EC.W2-6, EC.W13-3, and EC.W14-3 revealed that these phages contained 71, 125, 189, and 113 predicted open reading frames (ORFs), respectively. All these ORFs were located on the positive strand of the genome. There are no potential tRNA-coding genes in the genomes of these phages. Among all the ORFs identified in these phages, the predicted proteins have known potential functions related to various processes such as endolysin, holin, tail fiber protein, DNA replication/transcription/packaging, and cell lysis. On the other hand, the remaining ORFs have been classified as hypothetical proteins.

### 3.6 *In vitro* bactericidal activity of phages

*In vitro* bactericidal activity of phages against *E. coli* ATCC25922 at various MOIs (Supplementary Figure S4). Within 24 h, the OD_600_ value of the positive control increased continuously from 0.05 to 1.25, whereas that of the negative control remained unchanged. Phage treatment significantly inhibited *E. coli* ATCC25922 growth at all MOIs within the first 8 h. However, the OD_600_ values gradually increased after 8 h of phage treatment. After 24 h, there was a significant difference in the OD_600_ values compared to the positive control and a considerable difference in the MOI values. The OD_600_ values continued to rise gradually until 24 h. Although the bacterial counts for all MOIs exceeded 10^8^ CFU/mL at 24 h, there was a significant difference between MOI 10 and 0.001 compared to the positive control. Interestingly, MOI 10 exhibited better lytic activity in the first 12 h than other MOI values. However, after 24 h, an MOI of 0.001 resulted in higher lytic activity. These findings demonstrated that these phages could significantly inhibit bacterial growth at all MOIs, with the highest bactericidal activity observed at an MOI of 0.001 after 24 h.

### 3.7 Comparative evaluation of *in vitro* lytic activity of individual phage’s and phage cocktails

**Figure 5:**
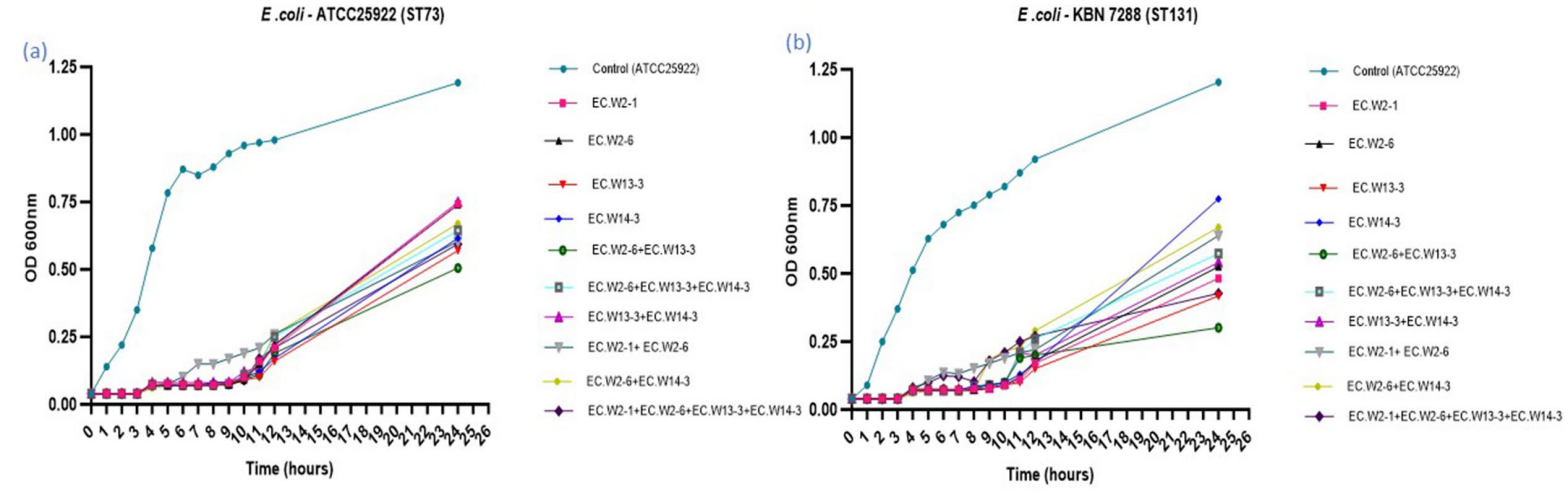
Lytic activity of four *E. coli* phages and their cocktail at MOI 0.001 against *E. coli* KBN 7288 (ST131) and *E. coli* ATCC 25922 (ST73).

This study compared individual phages and cocktails to determine their ability to inhibit bacterial growth in two *E. coli* strains (ATCC25922-ST73 and KBN7288-ST131). Analysis of bacterial growth curves over 24 h revealed that both individual phages and phage cocktails significantly inhibited *E. coli* ATCC25922 growth throughout the study. Specifically, from 0 to 12 h, the individual phages and cocktail significantly suppressed the bacterial growth. However, during the 6–10-h timeframe, the cocktail composed of EC.W2-1 and EC.W2-6 showed slightly lower inhibition than individual phages. Notably, after 24 h, the phage cocktail consisting of EC.W2-6 and EC.W13-3 showed a high level of bacterial growth inhibition (Figure 5a). Similarly, when evaluating the lytic activity of individual phages and the phage cocktail against *E. coli* KBN 7288 (ST131), no significant changes in growth inhibition were observed within the first 4 h. However, after 4 h, the phages displayed varying levels of lytic activity. Consistent with these findings, the phage cocktail consisting of EC.W2-6 and ECW13-3 exhibited high inhibition of *E. coli* KBN 7288 growth after 24 h (Figure 5b). This study also analyzed the lytic activity against *E. coli* KBN 7288 by using various MOI of phage cocktails and compared with their single phage, where the cocktail (EC.W2-6 and ECW13-3) at MOI 10 and MOI 1 provided better lytic activity against *E. coli* KBN 7288. However, there were no significant differences among the other MOIs of the phage cocktail (Supplementary Figure S5).

### 3.8 Evaluation of the efficacy of phages to treat *Escherichia coli* ST 131 *in vivo*

**Figure 6:**
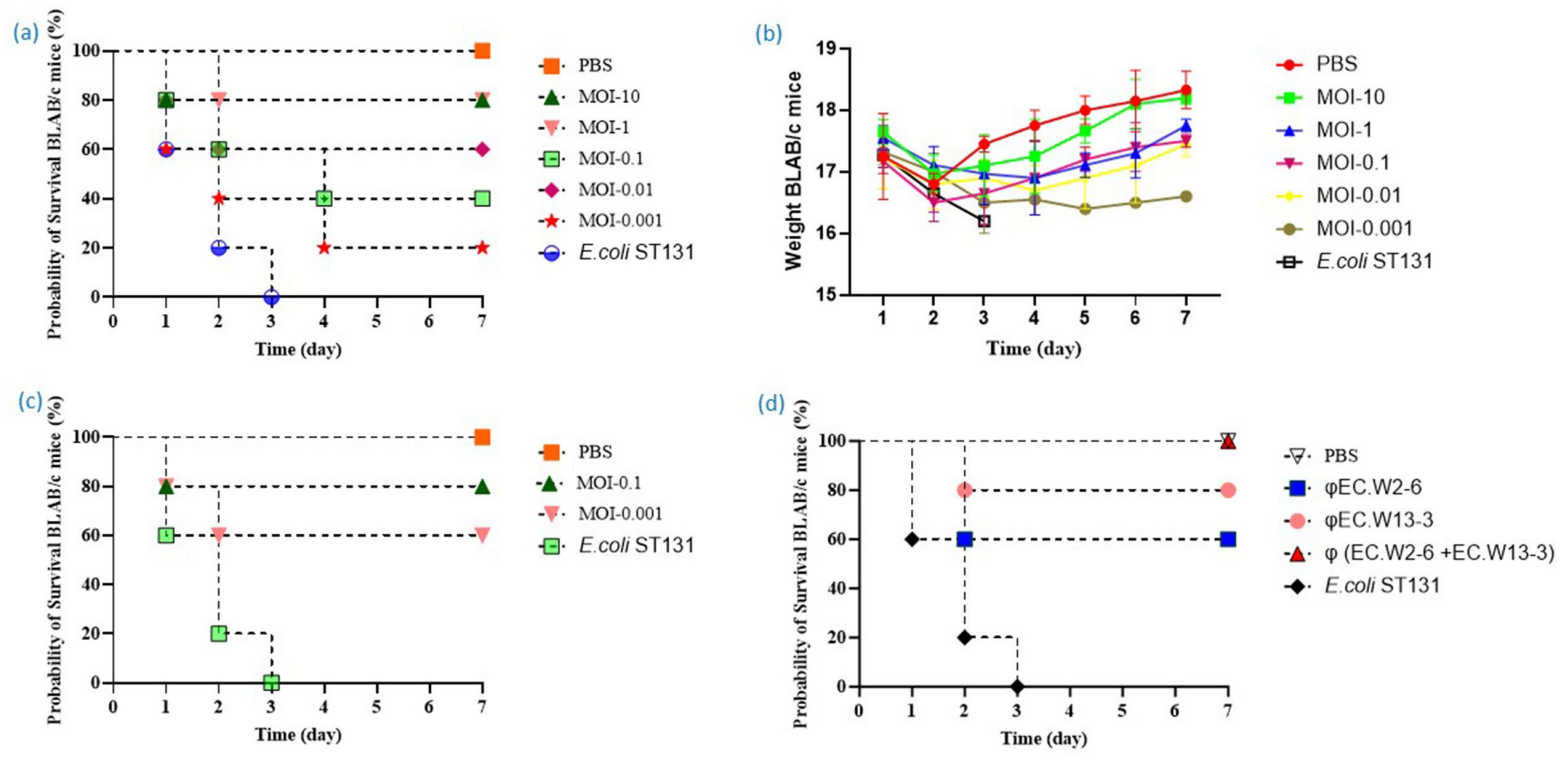
This study analyzes the toxicity of *E. coli* ST131 (KBN 7288) in BALB/c mice, identifies the optimal MOI for effective survival in phage therapy, and evaluates the efficacy of single phage and phage cocktails. (a) Survival curve of mice treated with phage EC.W13-3 at various MOIs or PBS. (b) Weight changes in mice one-week post *E. coli* administration. The weights of mice in the free phage group, mice treated with phage EC.W13-3 at various MOIs, and negative control groups were monitored for 2 week. (c) Evaluation of the efficacy of repeated phage therapy against *E. coli* ST131. The survival curve of mice given phage EC.W13-3 for 3 days at MOI-0.1 and MOI-0.001. (d) The study evaluates the efficacy of single phage and phage cocktail therapy against *E. coli* ST131 at MOI-0.1 in a mice model. The bacterial dose of 10^9^ CFU was injected at 0 h, and after 1 h, the mice were infected with 10^9^ PFU of phage.

**Figure 7:**
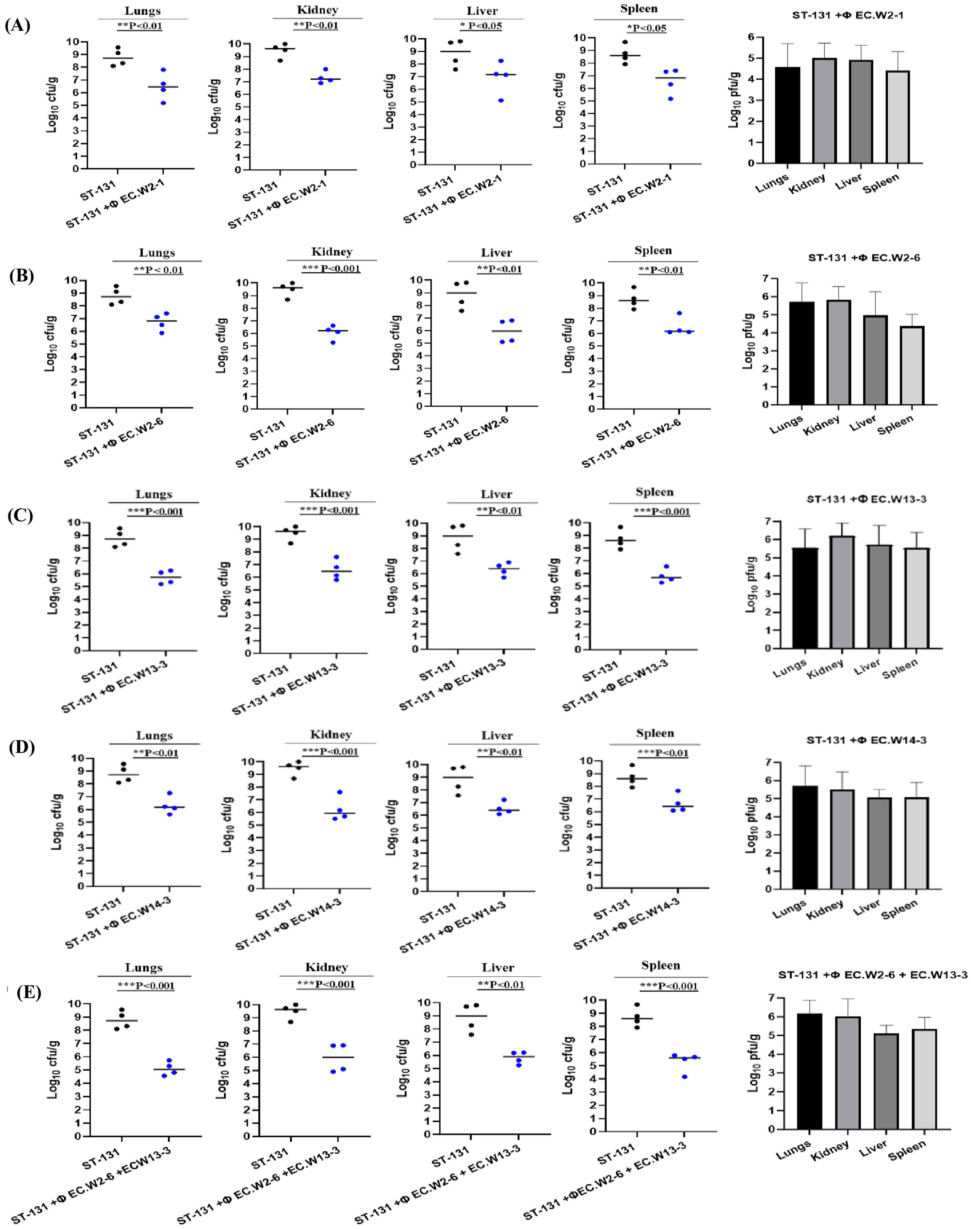
Phage therapy in a mouse model of ST131 MDR bacteraemia. BALB/c, 6-week-old female mice, were infected with an intraperitoneal (IP) injection of 10^9^ CFU of *E. coli* KBN7288. One hour post-infection, mice received an IP with 10^9^ PFU of four purified *E. coli* phages individually **(A)** EC.W2-1, **(B)** EC.W2-6, **(C)** EC.W13-3, **(D)** EC.W14-3, and as a cocktail **(E)** EC.W2-6 + EC.W13-3. Disease severity was evaluated, and organ samples were collected and plated to determine bacterial CFU and phage PFU. The efficacy of each treatment was assessed based on the CFU of *E. coli* KBN7288 in various organs. Black dots represent individual mice given bacteria alone, and blue dots represent the phage-treated, infected mice. Black bars represent the mean values for each group. The *p*-values were generated using a *T*-test, with one, two, or three stars denoting the significance level. One star (*) indicates *p* < 0.05, two stars (**) indicate *p* < 0.01, and three stars (***) indicate *p* < 0.001. Error bars denote standard deviation (*n* = 4).

The aim of this study was to assess the effectiveness of a phage-and-phage combination against *E. coli* ST131. Mice treated with phage EC.W13-3 at MOIs of 10 and 1 exhibited approximately 80% survival rates within 7 days. In contrast, the untreated positive control group experienced 100% mortality within 3 days, whereas the negative control group showed complete survival (Figure 6a). The study also monitored changes in the body weight of mice, revealing weight gains in mice treated with EC.W13-3 at MOIs of 10 and 0.01, indicating positive therapeutic effects. Conversely, mice exposed to a lower MOI of 0.001 or the control group displayed gradual weight loss, suggesting disease progression or ineffective treatment (Figure 6b). Additionally, a 3-day repeated treatment of EC.W13-3 at MOI 0.1 and MOI 0.001 significantly increased the survival rate by approximately 40% (Figure 6c). When investigating the effects of single phages EC.W2-6 and EC.W13-3 and their combination at MOI 01, the combination demonstrated a 100% survival rate in mice, whereas the survival rates for single phages were 60 and 80% (Figure 6d). This study evaluated the impact of individual phage treatments, including EC.W2-1, EC.W2-6, EC.W13-3, and EC.W14-3, on bacterial burden in various organs of a mouse model. Treatment with each phage substantially reduced the bacterial load across multiple organs, and phage counts (PFU/g) were calculated from the plaques on *E. coli* 10P0 KBN7288. Specifically, treatment with EC.W2-1 resulted in a significant reduction in bacterial load in the kidneys (2.5 log10 cfu/g), lungs (2.5 log10 cfu/g), liver (1.9 log10 cfu/g), and spleen (1.7 log10 cfu/g) (Figure 7a). Similarly, EC.W2-6 exhibited efficacy, demonstrating significant reductions in bacterial burden in the kidneys (3.2 log10 cfu/g), lungs (1.8 log10 cfu/g), liver (2.20 log10 cfu/g), and spleen (1.75 log10 cfu/g) (Figure 7b). Furthermore, treatment with EC.W13-3 resulted in notable reductions in log10 cfu/g, approximately 3 in the kidneys, 2.5 in the lungs, 2.55 in the liver, and 1.5 in the spleen (Figure 7c). In addition, EC.W14-3 significantly reduced log10 cfu/g by 2.7 in the lungs, 2.5 in the kidneys, 1.9 in the liver, and approximately 2.4 in the spleen (Figure 7d). Moreover, combining EC.W2-6 and EC.W13-3 in a cocktail showed enhanced effectiveness compared with individual phage treatments. This combination led to a remarkable reduction in bacterial burden in the kidneys (3.3 log10 cfu/g), lungs (3 log10 cfu/g), liver (2.5 log10 cfu/g), and spleen (2.5 log10 cfu/g) (Figure 7e).

## 4 Discussion

In this study, four *E. coli* phages (EC.W2-1, EC.W2-6, EC.W13-3, and EC.W14-3) were isolated in the hospital sewage water. Morphological features and sequence analysis revealed that phages have strong similarities to the *Straboviridae* and *Podoviridae* families, positioning them as promising candidates for phage-based therapies against *E. coli* infections because of their ability to effectively target and eliminate *E. coli* bacteria and combat antibiotic resistance (Koonjan et al., 2021; Wolfram-Schauerte et al., 2022). Our isolated phages displayed a broad host range, effectively targeting 94.44% (17/18) of *E. coli* ST131 isolates. Additionally, they exhibited lytic activity against 14 different ST types, including 60 MDR *E. coli* isolates, with lytic percentages ranging from 50.0 to 56.6% (Shamsuzzaman et al., 2024). The phages remained stable over a wide pH range (2–10) and at temperatures ranging from 4 to 60°C, indicating their ability to survive under various environmental conditions. The extremely low optimal incubation period and moderate burst size highlighted the high proliferation efficiency and lytic activity of these phages. In an *in vivo* experiment, phage treatment significantly reduced the bacterial load, making them potential candidates for phage therapy against *E. coli* ST131 and other ST types of *E. coli* (Dufour et al., 2016; Green et al., 2017).

Whole-genome analysis revealed that none of the four phages contained virulence, antibiotic resistance, or bacterial toxin-related genes, suggesting a minimal risk of promoting antibiotic resistance and making them suitable for treating bacterial infections (Chaudhary et al., 2022). Depolymerases are enzymes that are encoded by phages infecting encapsulated bacteria, typically within open reading frames linked to structural proteins, often found in tail fibers, base plates, and neck regions (Guo et al., 2023). The analysis of isolated phage genomes revealed the presence of several tail fiber proteins with a high degree of similarity, ranging from 94 to 100%, to those found in other *E. coli* phages (Supplementary Table S3). By recognizing specific ligands on bacterial surfaces, these depolymerases specifically target capsular polysaccharides (CPSs) and lipopolysaccharides (LPSs), resulting in the breakdown of their repeating units of polysaccharides (Cai et al., 2023). Instead of directly killing bacteria, phage depolymerase strips away the protective polysaccharide layers, rendering the bacteria vulnerable to the immune system and antibacterial agents (Topka-Bielecka et al., 2021). Based on this information, we can assume that these phages could produce these enzymes, resulting in the high killing ability of *E. coli* ST131 isolates. Phage EC.W2-6 is an interesting bacteriophage that contains an endolysin with a remarkable 98.77 and 96.93% similarity to the endolysins found in Escherichia phages (YP_001671762, YP_00920818). Endolysins are enzymes that target bacterial cell wall peptidoglycans and cause cell lysis (Murray et al., 2021). Their specificity, unique mode of action, and lack of resistance mechanisms make them promising alternatives to antibiotics, particularly for combating multidrug-resistant infections (Nelson et al., 2012). Analysis of phage genomes revealed several proteins potentially crucial for combating MDR *E. coli*, especially ST131. These include holins, membrane proteins forming pores to release phage progeny (Fernandes and São-José, 2016); putative peptidases, involved in protein breakdown (Matsumi et al., 2005); replication, recombination, and repair proteins, maintaining phage genome integrity (Poteete, 2004); and packaging machinery proteins for DNA packaging and phage assembly (Isidro et al., 2004). Baseplate proteins facilitate host recognition and infection (Leiman et al., 2003). While the roles of several hypothetical proteins remain unclear, a full understanding of these proteins is essential for optimizing phage therapy (Shang et al., 2023).

To use phages to treat human bacterial infections, translation from *in vitro* activity to *in vivo* efficacy is not guaranteed, despite a high success rate (Dufour et al., 2016). Our investigation into the potential of the four isolated phages revealed that they could infect targeted ST131 isolates and reduce the bacterial load in the survival of mice models. Using animal pneumonia, septicemia, and urinary tract infection models, isolated phages LM33_P1 and HP3 showed *in vivo* efficacy in reducing bacterial load in several organs (Dufour et al., 2016; Green et al., 2017). This study found that a combination of phages (phage cocktail) and repeated phage therapy was much more effective than a single-dose phage therapy. Repeated phage therapy using the EC.W13-3 phage at an MOI of 0.1 and MOI 0.001 significantly increased the survival rate. The survival rate increased from 40 to 80% at MOI 0.1 and from 20 to 60% at MOI 0.001. Additionally, using the phage cocktails EC.W2-6 and EC.W13-3 resulted in 100% survival, whereas single-phage therapy achieved 60 and 80% survival rates at MOI-01, respectively. A previous study showed that the application of phage cocktails and repeated phage treatments can remarkably reduce the bacterial load of nosocomial pathogens in hospital wastewater (Weissfuss et al., 2023). According to this study, phage treatment effectively reduced bacterial counts in different organs. Specifically, phages EC.W2-1 and EC.W2-6 were found to have lower bacterial counts in the kidneys, lungs, liver, and spleen. Additionally, phages EC.W13-3 and EC.W14-3 also showed significant reductions in the bacterial load. This study also found that combining EC.W2-6 and EC.W13-3 in a cocktail enhanced the effectiveness of treatment, resulting in reduced bacterial levels across all organs. Although these results are promising, it is essential to note that animal experiments have limitations and caution should be exercised when translating these findings to the clinical setting (Henry et al., 2013). Bacterial resistance to phage infection is a significant challenge (Örmälä and Jalasvuori, 2013), compounded by the impact of the human immune system (Łusiak-Szelachowska et al., 2014), including the production of anti-phage antibodies (Bearden et al., 2005). Previous research has shown that the vital antibody response to φX-174 serves as a crucial indicator of immune responsiveness in humans (Bearden et al., 2005), with significant implications for individuals with conditions such as HIV (Bernstein et al., 1985), genetic immunodeficiencies (Shearer et al., 2001) and those undergoing immunosuppressive treatments (Hayashi et al., 2018). In a clinical study involving immunocompromised 60% patients receiving phage therapy for bacterial infections, the local administration of phage resulted in low antibody neutralization in serum, suggesting potential efficacy in high-risk patient populations (Green et al., 2017).

This study demonstrates a significant advancement in phage therapy against MDR *E. coli* ST131. Four novel phages, isolated from hospital sewage, exhibited high individual lytic efficacy (>90%), surpassing the effectiveness of previously reported phages such as ΦLM33_P1 (70% efficacy against ST131 and ST69), ΦHP3, ΦEC1, and ΦCF2 (Dufour et al., 2016; Green et al., 2017). The increased efficacy of our phages is likely due to their greater potency and taxonomic diversity. Unlike earlier phages from the *Autographiviridae* and *Myoviridae* families, our phages belong to the *Straboviridae and Podoviridae* families, indicating distinct mechanisms of action and host interactions (Koonjan et al., 2021; Wolfram-Schauerte et al., 2022). Most importantly, our phage cocktail showed superior *in vitro* and *in vivo* performance, highlighting the promise of combination phage therapy as a more effective treatment for *E. coli* ST131 in clinical settings (Weissfuss et al., 2023).

## 5 Conclusion

Finally, this research highlights the serious global health threat of multidrug-resistant bacterial strains, specifically the *E. coli* ST131 clone. This study isolated four lytic phages (EC.W2-1, EC.W2-6, EC.W13-3, EC.W14-3) from hospital sewage water. These phages showed a broad host range and effectively lysed 94.4% of highly pathogenic *E. coli* ST131 isolates. They exhibited stability under different pH and temperature conditions and demonstrated strong lytic activity *in vitro*. Genomic analysis indicated the lack of virulence or drug-resistance genes. *In vivo* experiments confirmed that the phages effectively protected against MDR *E. coli* ST131 colon. In the injectional mice model, single phage and phage cocktail administrations improved survival rates and significantly reduced bacterial load in various organs. Based on this comprehensive evaluation, we propose phage EC.W2-1. EC.W2-6, EC.W13-3 and EC.W14-3 are potential solutions for targeting the ST131 pandemic. They provide hope amidst the growing threat of multidrug-resistant bacterial infections.

## Data Availability

The complete genome sequences of phages EC.W2-1, EC.W2-6, EC.W13-3, and EC.W14-3 have been deposited in GenBank with the NCBI accession numbers PP445228, PP445229, PP496997 and PP496998, respectively.

## Footnotes

^1^ https://btseq.celemics.com/

^2^ https://blast.ncbi.nlm.nih.gov/Blast.cgi

^3^ https://rast.nmpdr.org/rast.cgi

^4^ https://www.geneious.com/

^5^ http://lowelab.ucsc.edu/cgi-bin/tRNAscan-SE2.cgi

^6^ https://cge.cbs.dtu.dk/services/ResFinder/

^7^ https://cge.cbs.dtu.dk/services/VirulenceFinder/

^8^ https://www.genome.jp/viptree/

^9^ https://ggdc.dsmz.de/submit_victor_job.php

^10^ https://darlinglab.org/mauve/mauve.html
