# Supplementary material for: Bacteriophage as a novel therapeutic approach for killing multidrug-resistant Escherichia coli ST131 clone
Source: Front Microbiol. 2024 Dec 12;15:1455710. doi: 10.3389/fmicb.2024.1455710 (PMC11670814; doi:10.3389/fmicb.2024.1455710)
Supplement: Supplementary file 1 [file Data_Sheet_1.docx]

**Bacteriophage as a Novel Therapeutic Approach for Killing Multidrug-Resistant *Escherichia coli* ST131 Clone**

**Md Shamsuzzamn^1,2^_,_ Shukho Kim^1,2^ and Jungmin Kim^1,2*^**

^1^Department of Biomedical Science, School of Medicine, Kyungpook National University, Daegu, Republic of Korea

^2^Department of Microbiology, School of Medicine, Kyungpook National University, Daegu, Republic of Korea

***Corresponding author:** E-mail: minkim@knu.ac.kr; Phone: +82-53-420-4840; Fax: +82-53-427-5664.

**Present address:** Department of Microbiology, Kyungpook National University School of Medicine ADD. (41944) 680 gukchaebosang-ro, Jung-gu, Daegu, Republic of Korea.


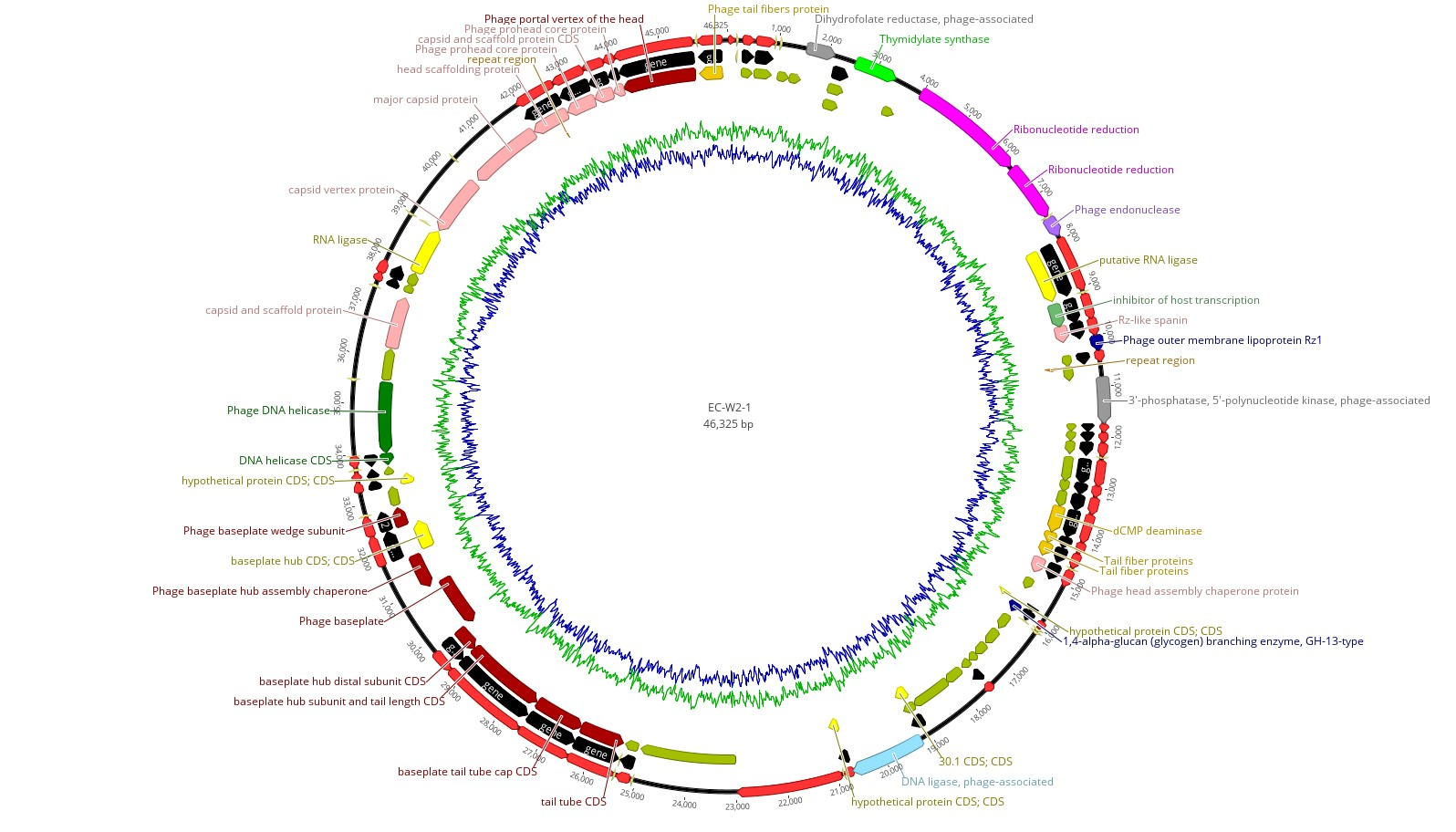


**Supplementary Figure S1(a).** Genomic feature maps of *E. coli* bacteriophages EC.W2-1.


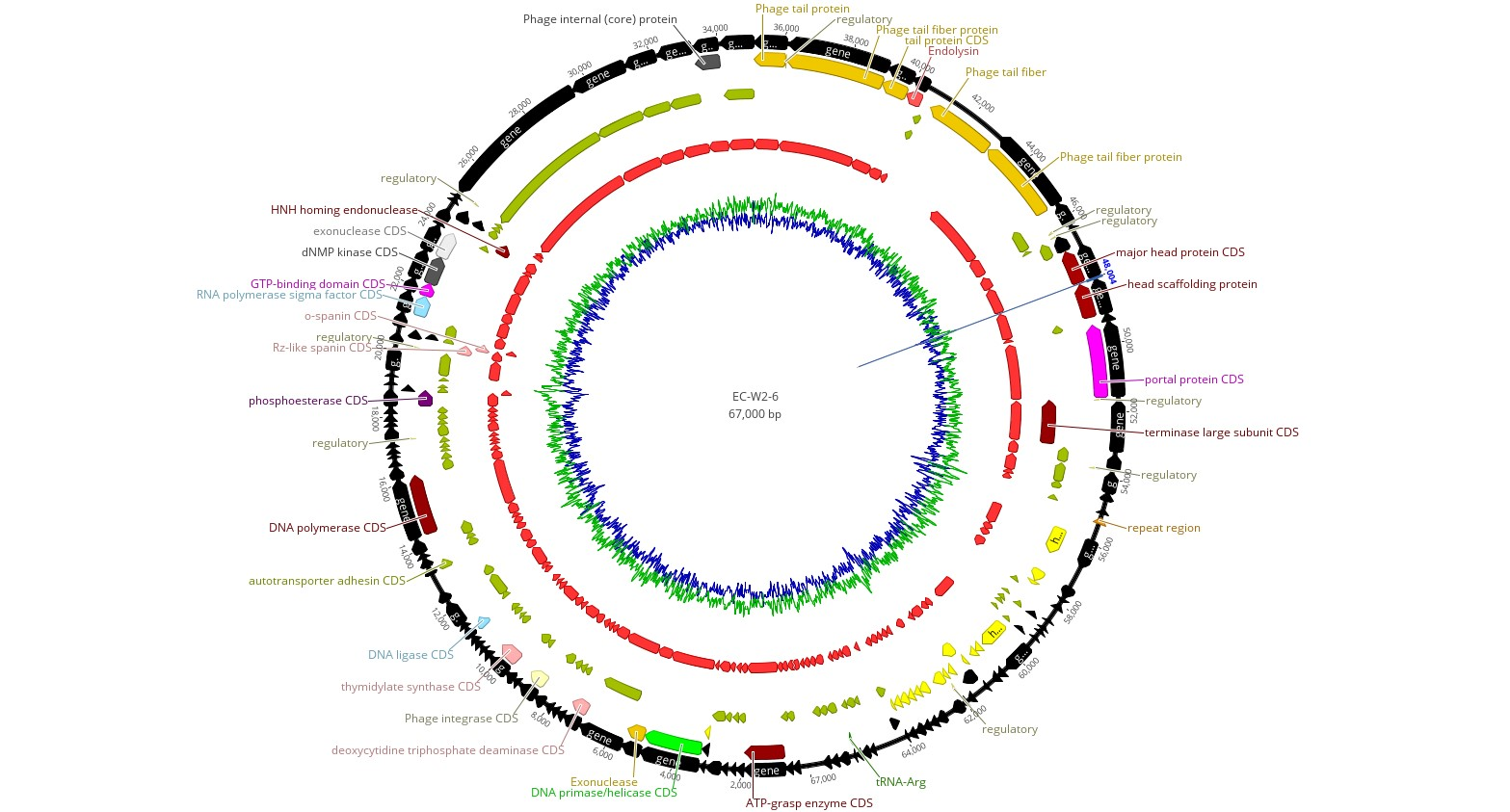


**Supplementary Figure S1(b).** Genomic feature maps of *E. coli* bacteriophages EC.W2-6.


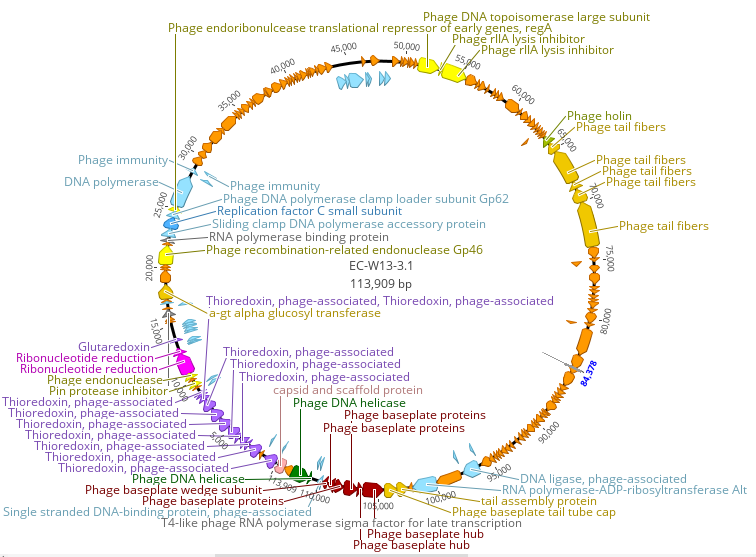


**Supplementary Figure S1(c).** Genomic feature maps of *E. coli* bacteriophages EC.W13-3.

**Supplementary**
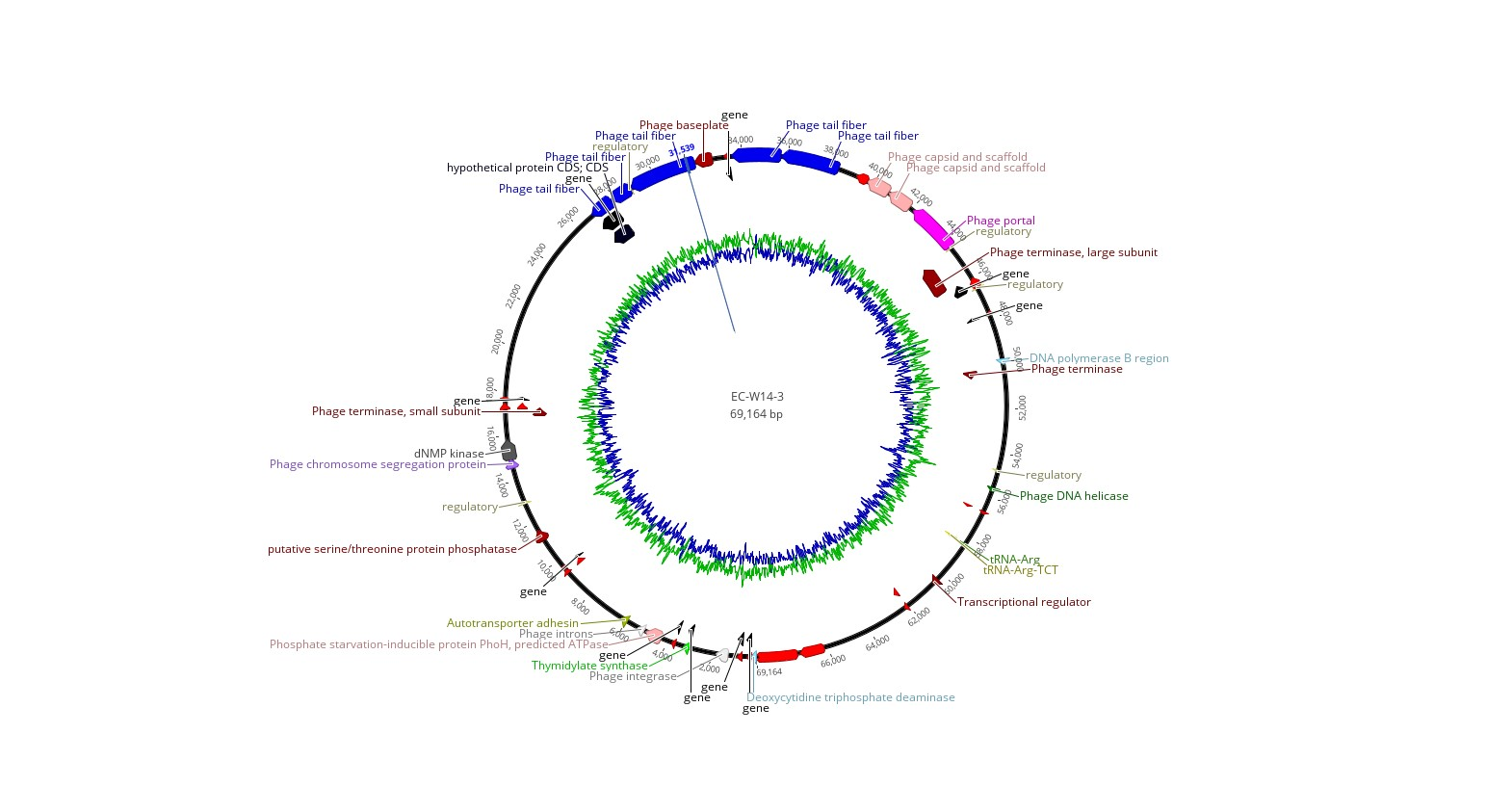
 **Figure S1(d).** Genomic feature maps of *E. coli* bacteriophages EC.W14-3.


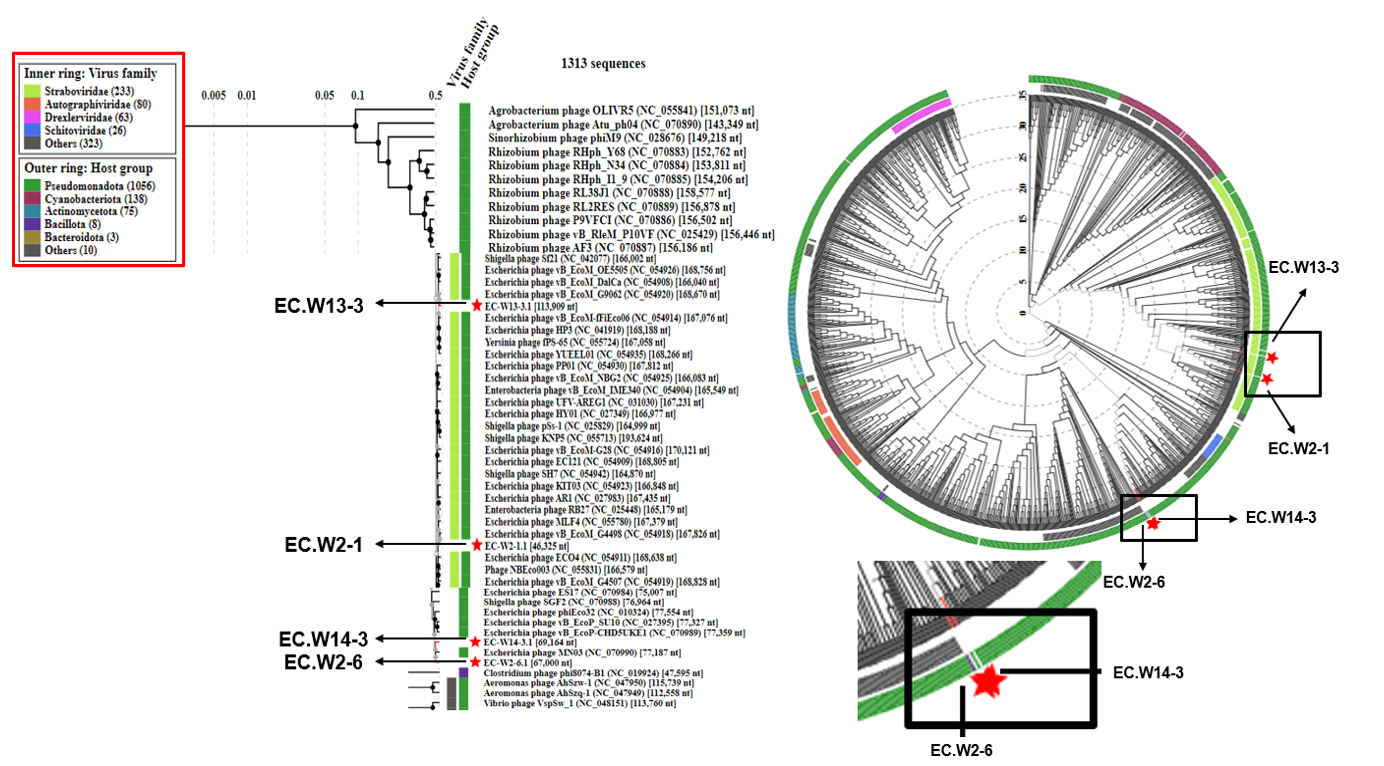
 **(a) (b)**

**Supplementary Figure S2.** Position of four *E. coli* phages in the phage proteomic tree. (a) The rectangular presentation shows the closest related phages to our isolates, indicated by a red asterisk. (b) Circular proteomic tree of prokaryotic dsDNA viruses, colour-coded by virus families and host taxonomic groups.


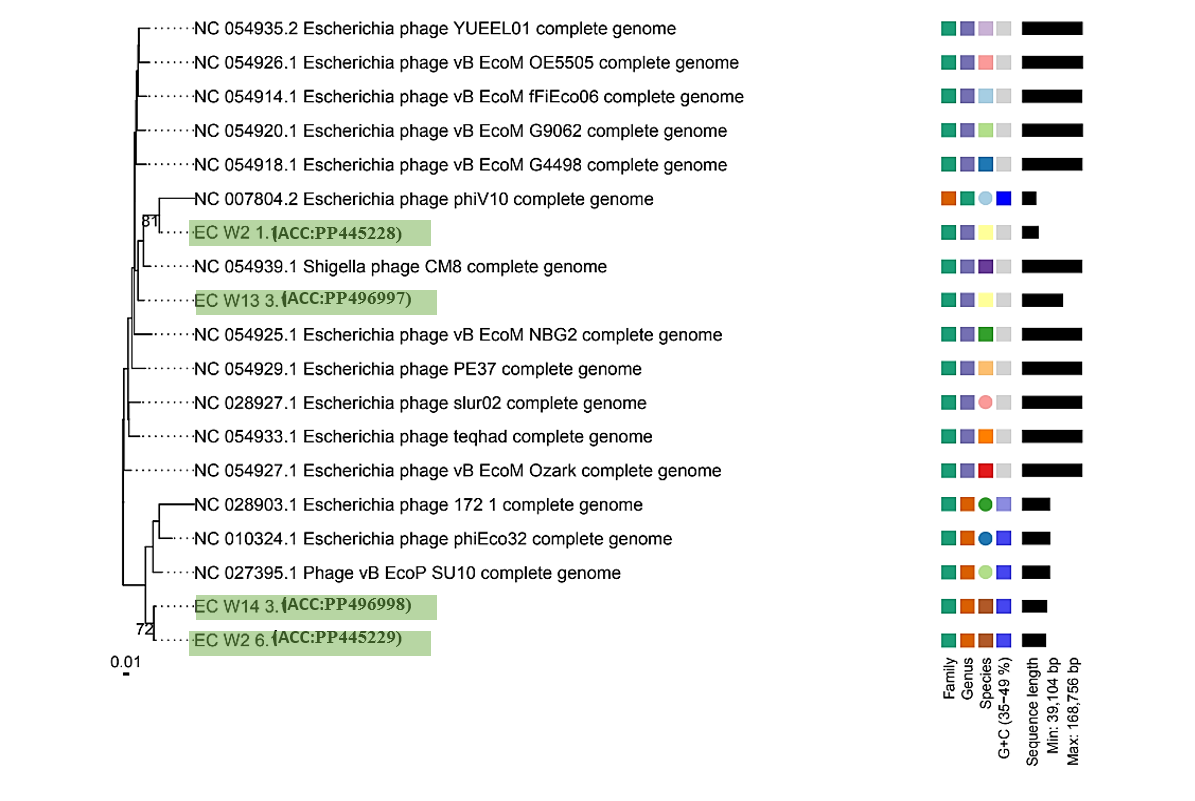


**Supplementary Figure S3.** Phylogenomic tree based on the whole genome sequence of phages—EC.W2-1, EC.W2-6, EC.W13-3, and EC.W14-3 reconstructed using TYGS.


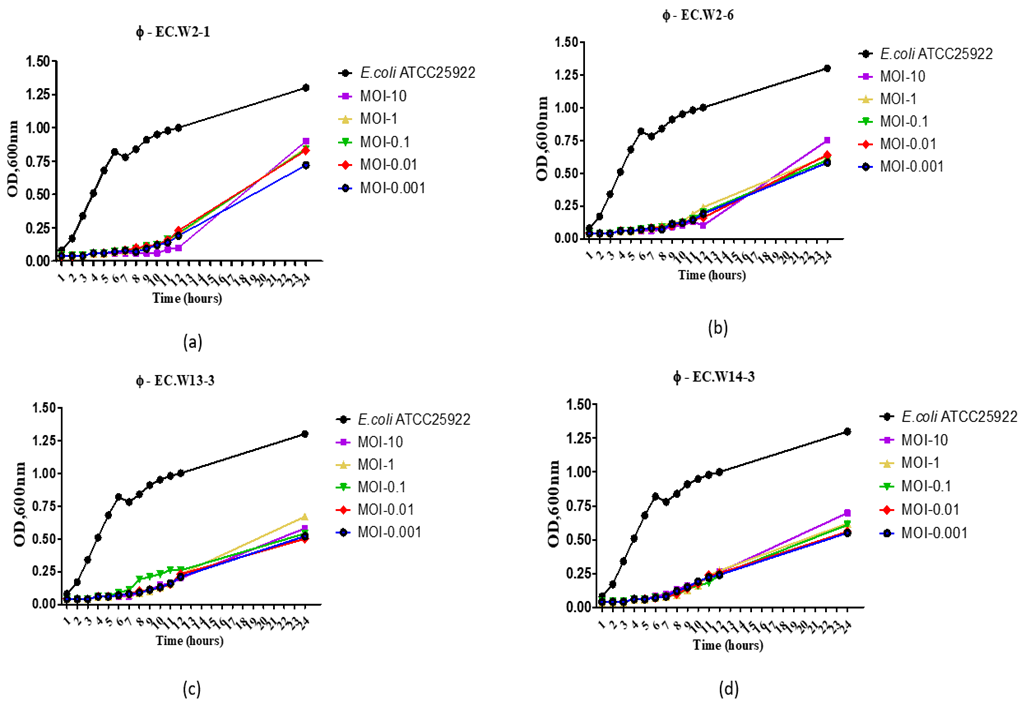


**Supplementary Figure S4.** *In vitro* bacterial lytic activities of four phages at various MOIs. Killing curves of *E. coli* ATCC25922 by using *E. coli* phages at MOIs of 10, 01 0.1,0.01 0.001, and for 24h. Each point represents the means ± SD of three replicate experiments.

**Supplementary Figure S5.** Comparison of the lytic activity of two novel *E. coli* phages and their cocktail at various MOI against *E. coli* KBN 7288 (ST131).

**Supplementary Table S1:** Calculated EOP values for each test phage against *E. coli* KBN 10PO7288 (ST131) compared to the reference phage, *E. coli* ATCC25922.

**Formula:**

**EOP** = Number of plaques formed by test phage / Number of plaques formed by reference Top of Form

| **Name of phage** | ***E. coli* ATCC25922** | **KBN 10PO7288** | **Efficiency of plating (EOP) values** |
| --- | --- | --- | --- |
| ΦEC.W2-1 | 2.3 x 10^-12^ | 5.9 x 10^-03^ | 2.565×10^9^ |
| ΦEC.W2-6 | 5.7 x 10^-13^ | 2.6 x 10^-06^ | 4.561×10^6^ |
| ΦEC.W13-3 | 6.5 x 10^-11^ | 1.7 x 10^-08^ | 261.538 |
| ΦEC.W14-3 | 4.3x 10^-13^ | 1.6 x 10^-07^ | 372.093 |

**Supplementary Table S2: Antimicrobial susceptibility of clinically isolated *Escherichia coli* ST131 clone**

| **Bacterial strains** | **Source** | **Amikacin** | **Gentamicin** | **Piperacillin** | **Trimethoprim** | **Cefoxitin** | **Cefazolin** | **Cefotaxime** | **Ceftazidime** | **Aztreonam** | **Cefepime** | **Ciprofloxacin** | **Imipenem** | **Meropenem** |
| --- | --- | --- | --- | --- | --- | --- | --- | --- | --- | --- | --- | --- | --- | --- |
| **KBN-10P00128** | **Urine** | **S** | **S** | **N/A** | **N/A** | **N/A** | **N/A** | **N/A** | **R** | **R** | **I** | **R** | **R** | **R** |
| **KBN-10P00238** | **Urine** | **N/A** | **S** | **N/A** | **N/A** | **N/A** | **R** | **R** | **N/A** | **N/A** | **R** | **S** | **R** | **R** |
| **KBN-10P01569** | **Urine** | **S** | **R** | **N/A** | **N/A** | **R** | **N/A** | **R** | **R** | **R** | **R** | **N/A** | **R** | **R** |
| **KBN-10P02048** | **Pus** | **S** | **S** | **N/A** | **N/A** | **R** | **N/A** | **R** | **R** | **R** | **R** | **N/A** | **R** | **R** |
| **KBN-10P02511** | **Pus** | **S** | **S** | **N/A** | **N/A** | **R** | **R** | **R** | **R** | **R** | **R** | **R** | **R** | **I** |
| **KBN-10P03005** | **Tissue** | **S** | **S** | **N/A** | **N/A** | **R** | **R** | **R** | **R** | **R** | **R** | **S** | **R** | **I** |
| **KBN-10P03440** | **Blood** | **I** | **R** | **S** | **R** | **I** | **R** | **R** | **R** | **R** | **R** | **S** | **S** | **I** |
| **KBN-10P03452** | **Bile** | **S** | **S** | **N/A** | **N/A** | **R** | **R** | **R** | **R** | **R** | **R** | **R** | **R** | **R** |
| **KBN-10P03979** | **Blood** | **R** | **R** | **N/A** | **N/A** | **R** | **R** | **R** | **R** | **R** | **R** | **R** | **S** | **S** |
| **KBN-10P05638** | **Urine** | **S** | **S** | **R** | **S** | **N/A** | **N/A** | **S** | **S** | **S** | **S** | **I** | **I** | **S** |
| **KBN-10P05702** | **Urine** | **S** | **R** | **N/A** | **S** | **R** | **R** | **R** | **R** | **R** | **R** | **R** | **I** | **R** |
| **KBN-10P05883** | **Urine** | **S** | **R** | **N/A** | **R** | **R** | **R** | **R** | **R** | **R** | **R** | **R** | **R** | **S** |
| **KBN-10P06658** | **Rectal swab** | **S** | **S** | **N/A** | **S** | **I** | **R** | **N/A** | **R** | **R** | **R** | **R** | **I** | **R** |
| **KBN-10P06781** | **blood** | **R** | **S** | **R** | **R** | **S** | **R** | **R** | **R** | **R** | **R** | **R** | **S** | **S** |
| **KBN-10P07282** | **blood** | **I** | **S** | **S** | **R** | **I** | **R** | **R** | **R** | **R** | **R** | **R** | **S** | **S** |
| **KBN-10P07288** | **blood** | **N/A** | **R** | **S** | **S** | **S** | **R** | **N/A** | **R** | **R** | **R** | **R** | **S** | **I** |

R=Resistant, S=Susceptible, I= intermediate and N/A =Not Available

**Supplementary Table S3.** Predicted tail protein of phages EC.W2-1, EC.W2-6, EC.W13-3 and EC.W14-3

| **Name of phage’s** | **Name of proteins** | **Positions** | **% similarity** | **References** |
| --- | --- | --- | --- | --- |
| **Φ**EC.W2-1 | Tail protein | 25,441 <- 26,406 | 100.00% | NC_054918 |
|  | Tail protein | 26,406 <- 27,500 | 100.00% | NC_054918 |
|  | Tail protein | 45,726 <- 46,217 | 100.00% | NC_041919 |
|  | Tail protein | 14,124 -> 14,360 | 100.00% | NC_041919 |
|  | Tail protein | 14,361 -> 14,669 | 100.00% | NC_054918 |
| **Φ**EC.W2-6 | Tail protein | 42,979 <- 45,537 | 99.30% | MT129653 |
|  | Tail protein | 40,770 <- 42,938 | 94.88% | [UEN68518.1](https://www.ncbi.nlm.nih.gov/protein/UEN68518.1?report=genbank&log$=prottop&blast_rank=1&RID=DT75CUYC013) |
|  | Tail protein | 39,121 <- 39,924 | 98.38% | NC_02739 |
|  | Tail protein | 42,979 <- 45,537 | 99.30% | MT129653 |
|  | Tail protein | 35,082 <- 36,050 | 99.48% | MT129653 |
| **Φ**EC.W14-3 | Tail protein | 26,802 <- 27,836 | 100.00% | MT129653 |
|  | Tail protein | 27,838 <- 28,806 | 100.00% | [UAG58402.1](https://www.ncbi.nlm.nih.gov/protein/UAG58402.1?report=genbank&log$=prottop&blast_rank=1&RID=DT4SK98E016) |
|  | Tail protein | 28,850 <- 31,867 | 99.30% | [UAG58401.1](https://www.ncbi.nlm.nih.gov/protein/UAG58401.1?report=genbank&log$=prottop&blast_rank=1&RID=DT4N1GGD016) |
|  | Tail protein | 33,526 <- 35,694 | 94.88% | [UEN68518.1](https://www.ncbi.nlm.nih.gov/protein/UEN68518.1?report=genbank&log$=prottop&blast_rank=1&RID=DT4GNNUE016) |
|  | Tail protein | 35,735 <- 38,293 | 99.65% | [YP_010674078.1](https://www.ncbi.nlm.nih.gov/protein/YP_010674078.1?report=genbank&log$=prottop&blast_rank=1&RID=DT49KH8B01N) |
| **Φ**EC.W13-3 | Tail protein | 103,382 <- 104,476 | 100.00% | [WP_171921440.1](https://www.ncbi.nlm.nih.gov/protein/WP_171921440.1?report=genbank&log$=prottop&blast_rank=1&RID=DT40654J013) |
|  | Tail protein | 69,982 <- 73,851 | 100.00% | [WHL25948.1](https://www.ncbi.nlm.nih.gov/protein/WHL25948.1?report=genbank&log$=prottop&blast_rank=1&RID=DT3XACUH013) |
|  | Tail protein | 68,858 <- 69,973 | 100.00% | [WP_171921502.1](https://www.ncbi.nlm.nih.gov/protein/WP_171921502.1?report=genbank&log$=prottop&blast_rank=1&RID=DT3U5KMH016) |
|  | Tail protein | 68,145 <- 68,795 | 100.00% | [WP_015983760.1](https://www.ncbi.nlm.nih.gov/protein/WP_015983760.1?report=genbank&log$=prottop&blast_rank=1&RID=DT3R8PA301N) |
|  | Tail protein | 65,188 <- 68,136 | 99.49% | [QXV85920.1](https://www.ncbi.nlm.nih.gov/protein/QXV85920.1?report=genbank&log$=prottop&blast_rank=1&RID=DT3HNC47013) |
